# Characterization and Bioactivity of Nanovesicles Recovered From Industrial Cheesemaking Whey Wastewater

**DOI:** 10.1111/1750-3841.71243

**Published:** 2026-06-23

**Authors:** Raffaella Latella, Anna Sansone, Carla Emiliani, Carla Ferreri, Letizia Mezzasoma, Rita Romani, Marco Gargaro, Roberto Maria Pellegrino, Husam B. R. Alabed, Eleonora Calzoni, Stefano Giovagnoli, Luana Lugini, Cristina Federici, Federica Fratini, Lorena Urbanelli, Sandra Buratta

**Affiliations:** ^1^ Department of Chemistry, Biology and Biotechnology University of Perugia Perugia Italy; ^2^ ISOF, Institute for Organic Synthesis and Photoreactivity, Consiglio Nazionale delle Ricerche Bologna Italy; ^3^ Centro di Eccellenza sui Materiali Innovativi Nanostrutturati (CEMIN) University of Perugia Perugia Italy; ^4^ Department of Medicine and Surgery University of Perugia Perugia Italy; ^5^ Department of Pharmaceutical Sciences University of Perugia Perugia Italy; ^6^ Department of Oncology and Molecular Medicine, Preclinical Research and Clinical Trials in Hematology and Oncology Unit Istituto Superiore di Sanità Rome Italy; ^7^ Core Facility Istituto Superiore di Sanità Rome Italy

## Abstract

Food‐derived nanovesicles (NVs), and particularly, those derived from milk, are considered promising nutraceutical candidates and potential carriers for drug delivery. Here, as an alternative to milk, we used a dairy byproduct as a source of NVs. Specifically, we isolated and characterized NVs from cheese whey wastewater (WWW) generated during the Grana Padano cheese production. The results of this study demonstrated that WWW‐NVs shared key features with milk‐derived vesicles in terms of biophysical properties (mean size <200 nm and negative zeta potential) and membrane lipid composition, characterized by the high content of phosphatidylcholine and sphingomyelin. WWW‐NVs were efficiently internalized by monocytic THP‐1 cells. In H_2_O_2_‐treated cells, WWW‐NVs significantly increased cell viability and reduced intracellular ROS levels to those of controls. Moreover, WWW‐NVs abrogated lipopolysaccharide and ATP‐induced inflammasome activation, as demonstrated by the suppression of caspase‐1 activation. Overall, WWW‐NVs exhibited biophysical and functional properties comparable to those reported for milk‐derived vesicles, while presenting advantages in terms of sustainability and waste valorization. The valorization of NVs from this industrial byproduct may bypass the need for pretreatment steps and avoid competition with the food chain. Therefore, WWW might represent a sustainable and alternative source of bioactive NVs, with promising applications in human health and biotechnology.

**Practical Applications:**

This study demonstrated that nanovesicles isolated from cheese whey wastewater, an industrial dairy by‐product, share morphological and biochemical properties with milk‐derived vesicles. The whey wastewater‐derived nanovesicles exerted antioxidant and anti‐inflammatory effects on mammalian cells. These findings support the potential use of dairy by‐products as an abundant, alternative, and sustainable source of bioactive nanovesicles avoiding competition with the food chain.

**Abbreviations**
ACN: acetonitrile
ANOVA: analysis of variance
DLS: dynamic light scattering
FAME: fatty acid methyl ester
FBS: fetal bovine serum
FDNV: food‐derived nanovesicle
GC: gas chromatography
GL: glycerolipid
IS: internal standard
LC‐MS: liquid chromatography‐mass spectrometry
MeOH: methanol
MTBE: methyl‐t‐butyl ether
MUFA: mono‐unsaturated fatty acid
NTA: nano tracking analysis
NV: nanovesicle
OD: optical density
PBS: phosphate‐buffered saline
PC: phosphatidylcholine
PE: phosphatidylethanolamine
PL: phospholipid
PS: phosphatidylserine
PUFA: poly‐unsaturated fatty acid
SEM: scanning electron microscopy
SFA: saturated fatty acid
SM: sphingomyelin
TG: triglycerides
Tol: toluene
WWW: whey wastewater

## 1 Introduction

Nanovesicles (NVs) represent a heterogeneous population of nanoparticles released by cells into the extracellular space and distributed throughout various biological fluids. NVs mediate intercellular communication by carrying molecules and signals, thus being responsible for physio‐pathological processes. NVs also mediate inter‐organism and cross‐species communication (J. Chen et al. 2024; Cao et al. 2019; Koeppen et al. 2021). Moreover, NVs derived from specific sources, such as milk, or those isolated from edible plants and fruits, possess intrinsic nutraceutical and therapeutic properties and can also serve as a platform for drug delivery (Kumar et al. 2024).

Based on such premises, the scientific community has recently focused its attention on food‐derived NVs (FDNVs), which are considered a promising nutraceutical tool, due to their ability to concentrate and carry specific biomolecules and nutrients (Rivero‐Pino et al. 2024). FDNVs are present in many biological sources, namely milk from different species, vegetables (e.g., ginger and garlic), fruits (e.g., grapefruit and lemon) (Nemati et al. 2022), and even probiotics (e.g., *Clostridium butyricum* and *Lactobacillus plantarum*) (Ma et al. 2022; Hao et al. 2021). The health‐promoting effects of FDNVs have been demonstrated in both in vitro and in vivo models (Turner 2024). Additional features, such as low toxicity, bioavailability, and stability within the digestive tract, make them promising and efficient bioactive nanocarriers (Turner 2024).

Among FDNVs, those isolated from milk of different species are the most extensively studied. Omics‐based approaches have provided a comprehensive characterization of the molecular cargo of milk‐derived NVs (Caira et al. 2025; Benmoussa et al. 2019; Buratta et al. 2023; van Herwijnen et al. 2016). In addition, these NVs exert anti‐inflammatory effects in vitro (Tong et al. 2023; Gao et al. 2024) and in a colitis murine model (Tong et al. 2021). Recent studies suggest that milk‐derived extracellular vesicles (EVs) can be engineered for the oral delivery of therapeutic peptides, showing injury‐targeting properties and significant cardioprotective effects in preclinical models of myocardial ischemia–reperfusion injury (Marsh, Amin, et al. 2025).

The agri‐food industry generates a variety of by‐products that are potentially harmful to the environment but also offer valuable opportunities for biotechnological innovation. Indeed, by‐products are often rich in natural bioactive compounds, which can be harnessed for various applications, including pharmaceuticals, cosmetics, and functional foods (Latella et al. 2024). Agri‐food by‐products represent valuable sources of bioactive compounds and, compared to primary raw materials, may also become a sustainable source of NVs. Vesicular encapsulation protects these molecules from degradation and may enhance their targeted delivery to specific cell types (Latella et al. 2024). Recently, NVs have been isolated from olive vegetation water; these NVs share several biophysical and biochemical features with edible plants‐ or fruits‐derived vesicles (Buratta et al. 2024).

NVs have also been successfully isolated from dairy by‐products, that is, from cheesemaking whey using different laboratory approaches (Sukreet et al. 2021; Del Saz‐Lara et al. 2025; Midtgaard et al. 2025).

In the present study, we isolated NVs from whey wastewater (WWW), the liquid byproduct generated during the industrial Grana Padano cheese production. NVs obtained from WWW (WWW‐NVs) were subjected to biophysical and biochemical characterization, with a specific focus on lipid composition, an aspect that has not been explored in previous studies. The results showed that WWW‐NVs shared biophysical and biochemical features with milk‐derived vesicles. Furthermore, we showed that WWW‐NVs are efficiently internalized by monocytic THP‐1 cells, where they exert cytoprotective, antioxidant, and anti‐inflammatory activities. These findings confirm that bioactive NVs can be directly isolated from an industrial dairy byproduct without requiring any pretreatment steps. These NVs presented a lipid composition and biological activities closely resembling those reported for milk‐derived vesicles.

## 2 Materials and Methods

### 2.1 Materials

Liquid chromatography‐mass spectrometry (LC‐MS)‐grade water (H_2_O), acetonitrile (ACN), methanol (MeOH), and isopropanol, ammonium fluoride, ammonium acetate, methyl‐t‐butyl ether (MTBE), chloroform (CHCl_3_), cis and trans fatty acid methyl esters (FAMEs), dimethyl disulfide, iodine, cholesterol, sphingomyelin, sodium thiosulphate, and iodine were purchased from Merck (Darmstadt, Germany). Toluene (Tol) was purchased from Carlo Erba (Milan, Italy). As internal standard (IS) for lipidomic analysis, we used Splash Lipidomix from Avanti Polar (Alabaster, AL, USA), whose composition has been previously reported (Buratta et al. 2024). Chloroform, methanol, isopropanol, diethyl ether, and n‐hexane (HPLC grade) were purchased from Millinckrodt (Phillipsburg, NJ, USA). POPC (1‐palmitoyl‐2‐oleoyl‐sn‐glycero‐3‐phosphocholine), POPE (1‐palmitoyl‐2‐oleoyl‐sn‐glycero‐3‐phosphoethanolamine), and POPS (1‐palmitoyl‐2‐oleoyl‐sn‐glycero‐3‐phosphoserine) were purchased from Larodan (Solna, Sweden). Cell culture reagents were from Euroclone S.p.A (Pero, Italy). Sapienic acid methyl ester, 8cis‐18:1 methyl ester, and sebaleic acid methyl ester were purchased from Lipidox (Lidingö, Sweden). Anti‐CD81, anti‐CD9, and anti‐calnexin antibodies were purchased from Santa Cruz Biotechnology (Dallas, Texas, USA).

### 2.2 Isolation of NVs From Industrial Cheesemaking Whey Wastewater

NVs were isolated from WWW, a byproduct of Grana Padano cheese production. This acid whey is obtained through acid coagulation during the fermentation used in Grana Padano cheese production. This byproduct is characterized by an acidic pH (∼5) and a lower protein content compared to sweet whey (Rocha‐Mendoza et al. 2021). WWW samples were collected and stored at −20°C until their use for NV isolation. NVs were isolated using a protocol previously employed for isolation of vesicles from milk (Caira et al. 2025; Özdemir 2020; Ross et al. 2021). WWW (50 mL) was centrifuged at 5000 × *g* for 30 min, and the resulting supernatant was diluted in phosphate‐buffered saline (PBS) (1:2, v/v) and subjected to centrifugation at 12,000 × *g* for 60 min using an F‐34‐6‐38 rotor in an Eppendorf 5804 R centrifuge (Hamburg, Germany). The supernatant was then centrifuged sequentially at 35,000 × *g* for 60 min and 70,000 × *g* for 60 min using a 70.1 Ti rotor (Beckman Coulture, Indianapolis, IN, USA), followed by filtration steps (0.45 and 0.22 µm). NVs were recovered by ultracentrifugation at 100,000 × *g* for 60 min using a Type 70.1 Ti rotor (Beckman Coulture).

### 2.3 SEM Analysis

The same protocol described in Buratta et al. (2024) was used to analyze WWW‐NVs. Briefly, various dilutions of WWW‐NVs fixed in glutaraldehyde were seeded onto glass coverslips. SEM images were obtained using a field emission LEO 1525 SEM (Zeiss, Thornwood, NY, USA) equipped with a Gemini column. The slide surface was coated with a layer of metal (Cr) (thickness ∼10 nm) using the Q150T ES‐Quorum high‐resolution sputter apparatus (24 s with sputter at 20 mA current intensity).

### 2.4 Nanoparticle Tracking Analysis

WWW‐NVs number and size distribution were analyzed with a NanoSight NS300 (Malvern Instruments, NanoSight Ltd., Salisbury, MD, USA) (Buratta et al. 2024). Each analysis was the integration of five 60‐seccon videos.

### 2.5 Dynamic Light Scattering Analysis

Dynamic light scattering (DLS) determined the zeta potential of WWW‐NVs by NanoBrook Omni Particle Size Analyzer (Brookhaven Instruments Corporation, USA) equipped with a 35‐mW red diode laser (nominal 640 nm wavelength) at 25°C (Aluigi et al. 2018; Cort et al. 2015). WWW‐NVs (50 µg of proteins) diluted with 1.5 mL of PBS was loaded into disposable cuvettes for DLS. The scattered light is then detected by the detector and translated to an autocorrelator. Five measurements have been acquired for each sample through Brookhaven Instruments Particle solutions v 3.6.0.6715.

### 2.6 Characterization of WWW‐NVs by Immunoblotting

WWW‐NVs (20–30 µg of proteins) were mixed with sample buffer 5X (1 M Tris‐HCl pH 6.8, 5% [w/v] SDS, 6% [v/v] glycerol, 0.01% [w/v] Bromophenol blue), with 125 mM DTT as the reducing agent. Proteins were separated by SDS‐PAGE and blotted onto polyvinyldene fluoride (PVDF) by semi‐dry transfer (Bio‐Rad, Hercules, CA, USA). Blocked membranes were incubated anti‐CD81, anti‐CD9, anti‐Tsg‐101, and anti‐calnexin antibodies overnight at 4°C. HRP‐conjugated secondary antibodies (GE Biosciences, Piscataway, USA) were detected using an ECL system (Life Technologies, Carlsbad, CA, USA).

### 2.7 Lipid Extraction and Q‐TOF LC/MS Analysis

The lipid extraction of WWW‐NVs (∼30 µg of proteins) was performed using the method published by Pellegrino et al. (2014). Briefly, an extraction mixture (MeOH/MTBE/CHCl_3_ 2:3:3, v/v) containing the IS (Splash Lipidomix) was added to NV samples. After vortexing, samples were centrifuged (16,000 × *g* for 10 min), and the supernatants were recovered and dried using a stream of nitrogen. Lipid extracts were resuspended in MeOH/Tol (9:1, v/v) and analyzed by UHPLC‐Q‐TOF (1260 Infinity II liquid chromatograph coupled with a 6530 Accurate‐Mass Q‐TOF mass spectrometer equipped with a JetStream source, all from Agilent Technologies, Santa Clara, CA, USA). Lipid classes were separated using a Waters Acquity CSH C18 UPLC column (Waters Corporation, Milford, MA, USA; dimensions: 100×2.1 mm, particle diameter: 1.7 µm) using the chromatographic conditions reported by Buratta et al. (2024). The mass spectrometric analysis, annotation, and quantification of lipid molecular species were performed according to Buratta et al. (2024). At the end of the workflow, a data table reported the concentration in nmol/µg proteins of the annotated lipids belonging to different lipid classes was obtained.

### 2.8 Fatty Acid Analysis by Gas Chromatography and GC‐MS

The fatty acid (FA) composition of WWW (1 mL) and WWW‐NVs (∼100 µg of proteins) was determined by isolation of the lipid fraction, transformation of the FA‐containing lipids into the corresponding fatty acid methyl esters (FAME), and analysis by gas chromatography (GC), according to previously published procedures (Ferreri et al. 2020; Küçüksayan et al. 2022). In Figure S1, two representative GC chromatograms show the FAME separation from WWW (panel A) and WWW‐NVs (panel B). To unambiguously assign the double bond position of unsaturated FA residues, the FAME mixtures obtained from WWW and WWW‐NVs were DMDS‐derivatized and analyzed by GC‐MS, as previously reported (Scanferlato et al. 2019; Tabolacci et al. 2025).

### 2.9 Untargeted Analysis of Polar Metabolites

Polar metabolites were extracted according to Cajka et al. (2023). Briefly, WWW‐NVs were mixed with MeOH/MTBE (1:5, v/v). After shaking, 1 vol of 10% MeOH (v/v) was added. The polar phase (200 µL) recovered by centrifugation was evaporated. The extracts resuspended in ACN/H_2_O (4:1, v/v) were analyzed by UHPLC‐Q‐TOF as previously described (Buratta et al. 2024). The analysis and annotation of MS data were carried out as reported in a previous study (Buratta et al. 2024). Following the data processing procedure, a data table reporting each annotated peak and its corresponding area was obtained.

### 2.10 THP‐1 Cell Cultures

THP‐1 human monocyte cells were maintained in RPMI 1640 medium with 10% (v/v) heat‐inactivated fetal bovine serum (FBS), 2 mM l‐glutamine, 1 mM sodium pyruvate, non‐essential amino acids, and antibiotics (100 µg/mL streptomycin and 100 U/mL penicillin) in 5% CO_2_ at 37°C. In every experiment including NVs treatment, THP‐1 cells were collected, suspended in fresh medium containing 10% of exosome‐depleted FBS (v/v), and seeded at a proper density onto plastic culture plates.

### 2.11 Cell Viability

Cell viability was assessed by MTT (3‐(4,5‐dimethylthiazol‐2‐yl)‐2,5‐diphenyltetrazolium bromide) assay. Note that 1 × 10^4^ THP‐1 cells were seeded in 96‐well plates and then WWW‐NVs (5 µg proteins) and/or H_2_O_2_ (250 µM) were added for 24 h at 37°C. At the end of incubation, cells were treated for 3 h with 5 mg/mL MTT solution. DMSO was used to solubilize formazan crystals. Absorbance was measured at 570 nm with a Beckman Coulter DTX880 microplate reader. The viability of treated cells was elaborated by comparing their optical density (OD) with that of control cells (OD of treated wells/OD of control wells) × 100.

### 2.12 Evaluation of NV Internalization by THP‐1 Cells

The uptake of WWW‐NVs by THP‐1cells was evaluated by assessing the intracellular localization of NVs labeled with DiL (1,1'‐dioctadecyl‐3,3,3',3'‐tetramethylindocarbocyanine perchlorate; Thermo Fisher Scientific, Carlsbad, CA, USA) by fluorescence microscopy and flow cytometry. Briefly, the supernatant recovered from the 12,000 × *g* centrifugation step was supplemented with DiL (50 µM). After incubation (30 min at room temperature [RT]), the supernatant was submitted to the centrifugation and filtration steps as described above. For fluorescence microscopy, THP‐1 cells (1 × 10^4^) were treated with DiL‐labelled NVs for 2 h. Cells were then rinsed in PBS, mounted, and analyzed with a Zeiss Axio Observer Z1 equipped with Apotome and digital Camera (Axiocam MRm; Zeiss, Oberkochen, Germany). The images are representative of one out of three separate experiments (Magnification 40×). Cells were fixed in 4% paraformaldehyde and F‐actin was labelled using fluorescein isothiocyanate (FITC)‐conjugated phalloidin (1:250) for 30 min, while cell nuclei were counterstained with 4′,6′‐diamidino‐2‐phenylindole. Subsequently, cells were examined using a Zeiss Axio Observer Z1 microscope equipped with an Apotome system and a digital camera (Axiocam MRm; Zeiss). Images shown are representative of one of three independent experiments (40× magnification).

The percentage of DiL‐labeled NV–positive cells was measured by flow cytometric analysis. Samples were run on LSR‐Fortessa (BD Biosciences) flow cytometer and analyzed using the FlowJo analysis software (Tree Star). Each experimental point was performed in triplicate.

### 2.13 Determination of Antioxidant Activity of WWW‐NVs

DCFH‐DA (2′,7′‐dichlorofluorescin diacetate) assay was used to evaluate intracellular ROS levels. Note that 1 × 10^4^ THP‐1cells were seeded in a 96‐well black plates, then incubated for 24 h with H_2_O_2_ (250 µM) without or with WWW‐NVs (5 µg proteins). Following incubation, plates were centrifuged (500 × g for 5 min) and treated for 1 h with DCFH‐DA (200 µM), collected by centrifugation and resuspended in PBS. DCF (dichlorofluorescein) fluorescence intensity was measured at excitation/emission wavelengths of 490/520 nm, with a Beckman Coulter DTX880 microplate reader. Data were calculated as percentage of DCF fluorescence with respect to control and normalized to cell viability evaluated by MTT assay.

### 2.14 Determination of Anti‐Inflammatory Activity of WWW‐NVs

THP‐ 1 cells (3×10^6^) were seeded in 12‐well culture dishes and treated for 1 h WWW‐NVs (2.5–10 µg proteins) and then primed with 10 µg/mL LPS for 20 min and activated with 5 mM ATP for 40 min (Nunzi et al. 2023).

THP‐1 cell lysates (15 µg of proteins), prepared using RIPA buffer containing protease and phosphatase inhibitors, were separated by 12% SDS‐PAGE, transferred on nitrocellulose membrane, and blocked in Roti‐Block (Roth GmbH, Germany) for 1 h at RT. The membranes were incubated overnight at 4°C with the following anti‐human Abs: anti‐NLRP3 (D4D8T) rabbit monoclonal antibody (#15101) and anti‐Caspase‐1 rabbit polyclonal antibody (#2225) (Cell Signaling Technology). After washing with TBS‐T (Tris buffered saline containing 0.1% Tween 20), membranes were incubated for 2 h at RT with HRP‐conjugated secondary Abs and detected using ECL system (Amersham Pharmacia Biotech). Anti‐β‐actin mAb (I‐19) antibody (Santa Cruz Biotechnology) was used for normalization after stripping. Densitometric analyses were carried out with the ImageJ software (https://imagej.nih.gov/ij/).

### 2.15 Statistical Analysis

The GraphPad Prism 5.0 software (GraphPad Software, Inc., San Diego, CA, USA) or R software (version 3.6.1; R Foundation for Statistical Computing, Vienna, Austria) were used to conduct statistical analyses. Data are presented as mean ± standard error (SE). The number of independent experiments (biological replicates, n) is specified in the figure legends. Comparisons were carried out with either Student's *t*‐test or a non‐parametric test, such as one‐way analysis of variance (ANOVA) with Dunnett's multiple comparison test or ANOVA with Tukey's. Parametric tests were applied based on their common use in similar exploratory studies on EVs and the assumption of approximate normal distribution of biological replicates. A *p* value below 0.05 was considered statistically significant.

## 3 Results and Discussion

### 3.1 Characterization of WWW‐NVs

**FIGURE 1:**
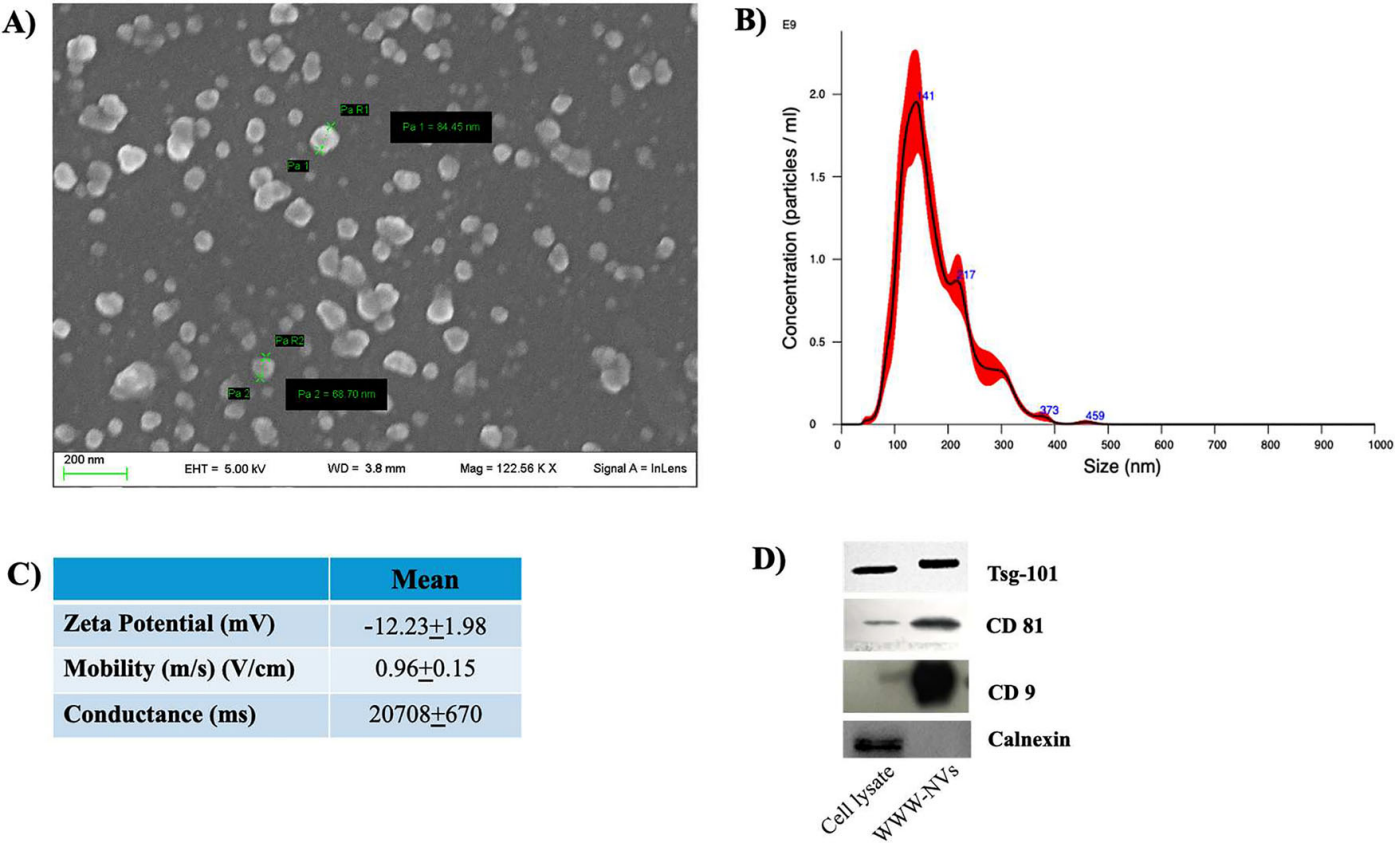
Characterization of the NVs isolated from WWW. (A) Representative image of WWW‐NVs visualized by SEM. The scale bar corresponds to 200 nm. (B) Concentration and size distribution of WWW‐NVs evaluated by NTA. The graph represents one of two independent experiments. (C) Zeta potential has been measured by DLS. Data are expressed as mean ± SD (*n* = 5). (D) Western blotting analysis using specific positive (Tsg‐101, CD81, and CD9) and negative (calnexin) vesicular protein markers. To assess the immunoreactivity of antibodies, proteins from BEAS‐2B lysates (30 µg of proteins) were separated in the same gel and subsequently electroblotted. Immunoblots are representative of other two independent experiments.

NVs were isolated from the WWW derived from Grana Padano cheese production using a protocol already employed for the isolation of NVs from milk (Caira et al. 2025). SEM images show that WWW‐NVs have size and morphology similar to NVs isolated from milk (Morozumi et al. 2021) (Figure 1A). Nano tracking analysis (NTA) measurements showed that the diameter of WWW‐NVs ranged from 100 to 300 nm (Figure 1B), with a mean mode value of 142 ± 3 nm (n = 2). This size distribution is consistent with that reported for vesicles isolated from cheesemaking whey (Del Saz‐Lara et al. 2025).

NVs recovered from 50 mL of WWW contained 104 ± 26 µg of protein, and the particle number quantified by NTA was 2.49 × 10^11^ (±2.05 × 10^10^). The NV yield from WWW was lower than that reported for raw milk, both in terms of µg of protein and number of NVs per mL of starting material (Grossen et al. 2021; Blans et al. 2017; Morozumi et al. 2021). This observation aligns with the findings of Sukreet et al. (2021), who also reported that whey yields a lower amount of small EVs compared to milk. In their case, this difference was attributed to the lower abundance of small EVs in acidic whey compared to milk and to the different isolation methods employed, as EVs from whey were isolated by tangential flow filtration, whereas EVs from milk were isolated by ultracentrifugation (Sukreet et al. 2021). Here, we did not directly compare WWW‐NVs with milk‐derived EVs, but isolated bioactive vesicles from an agro‐industrial byproduct routinely obtained during industrial Grana Padano cheese production, instead of a nutritionally valuable source such as milks. The ratio between NV number and proteins was about 10^9^ vesicles/µg of proteins, consistent with NVs previously isolated from raw bovine (Morozumi et al. 2021) and donkey milk (Caira et al. 2025).

Based on the yields obtained per milliliter of starting material in our experimental conditions, we calculated that the NV yield was ∼5 × 10^12^ per liter and ∼2 mg proteins per liter; this value is lower than that reported for milk‐derived EVs (>10^14^ particles per liter) (Marsh, Beard, et al. 2025). Although this value is indicative and dependent on processing parameters, it might represent an indication for future large‐scale NV production.

DLS analysis showed that WWW‐NVs had a negative zeta potential (Figure 1C), consistent with NVs isolated from bovine milk (Matic and Dia 2022) and whey (Del Saz‐Lara et al. 2025). Finally, immunoblotting analysis revealed that WWW‐NVs were positive to vesicular markers CD81, CD9, and Tsg‐101, whereas the negative marker calnexin was absent (Figure 1D). To exclude contamination by abundant milk proteins such as caseins, SDS‐PAGE analysis was performed, loading purified casein in a separate lane as a control. Proteins were transferred onto PVDF membranes and stained with Ponceau Red. As shown in Figure S2, no bands corresponding to caseins were detected in the NV samples. Consistently, a previous study demonstrated that cheesemaking whey represents a sustainable source of EVs with reduced casein contamination compared to milk (Del Saz‐Lara et al. 2025). In that study, EVs were isolated by ultracentrifugation and, for comparison, by TFF and polythylene glycol (PEG) precipitation to evaluate methods suitable for pharmaceutical scale‐up. The authors concluded that additional purification steps are required to achieve optimal EV purity and that acidification effectively reduces protein contaminants in milk, whereas it has minimal impact in whey.

Overall, these findings indicated that NVs recovered from the WWW have cup‐shaped morphology, mean size (<200 nm), and negative zeta potential similar to small EVs derived from bovine milk (Samuel et al. 2017; Samuel et al. 2023; Kankaanpää et al. 2024; Matic and Dia 2022) and cheesemaking whey (Sukreet et al. 2021; Del Saz‐Lara et al. 2025). In our NV preparations, we also detected CD81, CD9, and Tsg‐101, which are considered positive markers of EVs (Welsh et al., 2024). These vesicular markers were not detected in EV fractions isolated from whey by TFF (Sukreet et al. 2021), whereas Tsg‐101 and CD63 were found in EVs isolated by cheesemaking whey (Del Saz‐Lara et al. 2025). It is well known that different isolation methods yield EV preparations enriched in specific EV subpopulations, which may differ in their protein marker composition (Li B et al. 2025). Taken together, our results suggest that the physicochemical characteristics of WWW, such as its lower protein content compared to milk (Rocha‐Mendoza et al. 2021), the processing steps (i.e., whether the whey fraction is fermented), and the vesicle isolation methods (Marsh et al. 2021), influence both the yield and the properties of the isolated NVs.

### 3.2 Lipid Composition of WWW‐NVs

**FIGURE 2:**
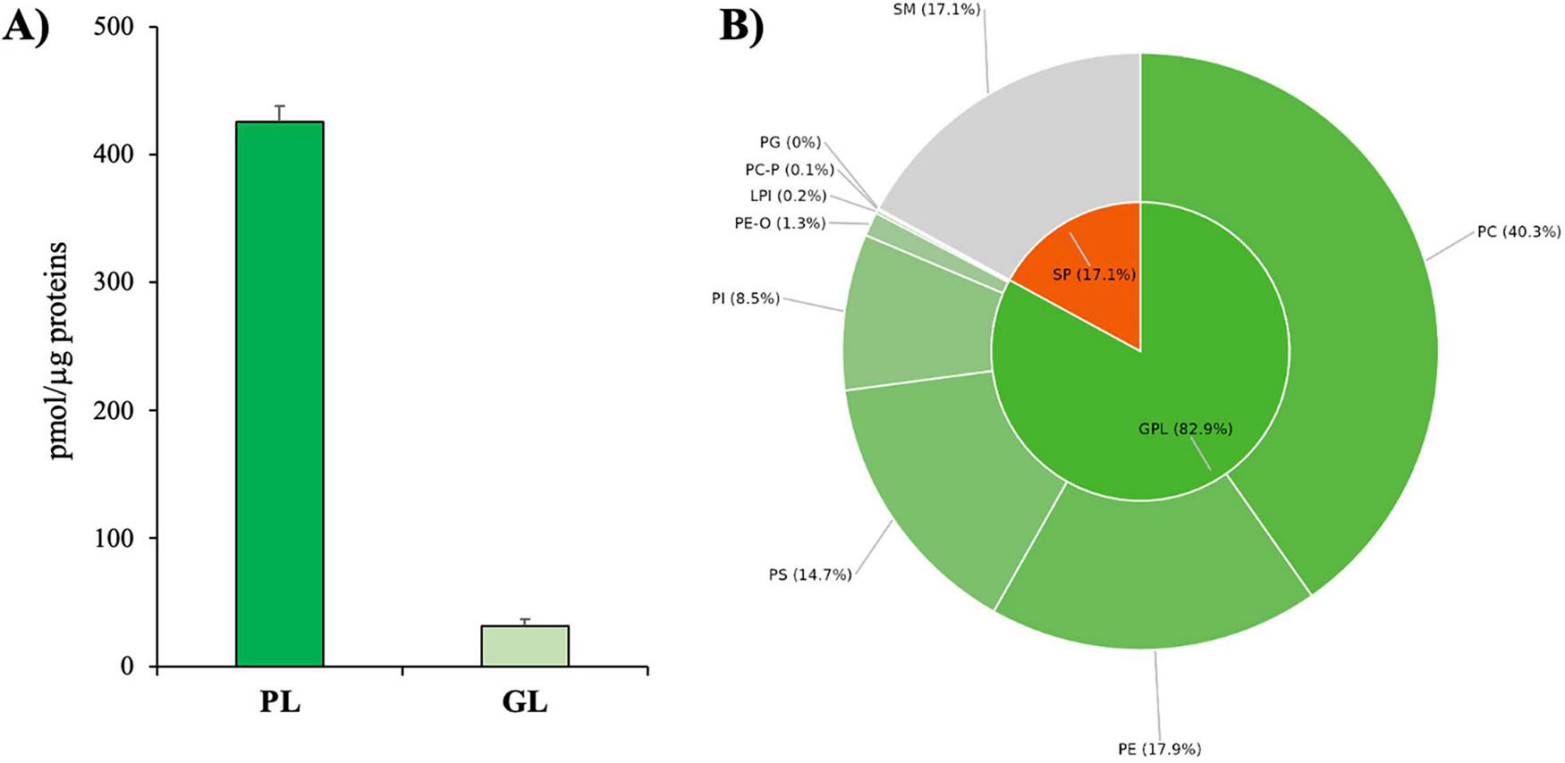
Lipid composition of WWW‐NVs. Lipids extracted from WWW‐NVs were analyzed by LC‐MS. (A) The graph shows phospholipid (PL) and glycerolipid (GL) content expressed as pmol/µg of proteins. The data are reported as mean ± S.E (*n* = 3). (B) The pie chart shows the amount of each PL class expressed as percentage of the sum of all identified PL species. Pie chart and the percentage values were generated using LipidOne 2.3 (Alabed et al. 2024).

The biochemical characterization of WWW‐NVs focused on the analysis of their lipid content, as this aspect has not yet been investigated in NVs isolated from dairy by‐products. In particular, the lipid class distribution and the total lipid FA profiles were evaluated by LC‐MS and GC, respectively. WWW‐NVs exhibited a higher phospholipid (PL) content, compared with glycerolipids (GL) (Figure 2A). Noteworthy, all annotated GL species belonged to triglycerides (TG). A low amount of GL compared to PL was also observed in NVs isolated from human and bovine milk (Blans et al. 2017; Grossen et al. 2021). Interestingly, the high PL/GL ratio suggests a low level of contamination by lipoprotein‐like particles or milk fat globules, which are typically enriched in TG (Buratta et al. 2023), thereby supporting an enrichment of vesicular structures in our NV preparations. Regarding PL, glycerophospholipids and sphingolipids accounted for 82±1.9% and 17±4.3%, respectively. PL was mainly composed of phosphatidylcholine (PC), followed by phosphatidylethanolamine (PE), sphingomyelin (SM), phosphatidylserine (PS), and phosphatidylinositol (Figure 2B). The high level of PC, PE, and SM appears to be a feature that WWW‐NVs shared with bovine milk‐derived vesicles (Blans et al. 2017; Kankaanpää et al. 2024). In NVs isolated from dromedary milk, the most abundant phospholipid was PC, followed by PE and PS (Yassin et al. 2016). These findings indicate that in terms of lipid class composition, WWW‐NVs closely resemble vesicles isolated from milk of different species. The enrichment in PC and SM observed in WWW‐NVs is consistent with the membrane organization typical of EVs derived from mammalian cells (Ghadami and Dellinger 2023). This lipid composition seems to contribute to membrane stability and may also influence interactions with recipient cells. Although direct relationships remain difficult to establish, accumulating evidence suggests that EV lipid composition plays a key role in modulating their uptake and biological activity (Skotland et al. 2019; Ghadami and Dellinger 2023).

Then, we evaluated the total lipid FA composition of WWW‐NVs, and unprocessed WWW for comparison. This aspect is particularly relevant because the types of FA esterified within membrane lipids modulate the physico‐biological features of the membrane. In addition, the saturation level of membrane PL is known to affect both the extracellular stability of NVs (Alvarez‐Erviti et al. 2011; Subra et al. 2007) and their targeting properties (Parolini et al. 2009; Buratta et al. 2021). In WWW‐NVs, saturated fatty acids (SFA) were ∼55% of total FA, while monounsaturated FA (MUFA) and polyunsaturated FA (PUFA) accounted for ∼30% and 9%, respectively (Table 1). In unprocessed WWW, SFAs (∼62%) were the most abundant, followed by MUFAs (∼30%) and PUFAs (∼5%). In both cases, palmitic (16:0) and stearic (18:0) acids were the most represented SFA, whereas the most abundant PUFA belong to the n‐6 series (Table 1). These data indicate that WWW‐NVs contain a higher content of PUFA‐ and a lower amount of SFA‐containing lipids compared with unprocessed WWW. Interestingly, NVs isolated from human milk are particularly enriched in PL species containing MUFA and PUFA (W. Chen et al. 2021).

**TABLE 1:** Fatty acid (% relative quantification), identified as fatty acid methyl esters (FAME), and corresponding fatty acid indexes of the fatty acid–containing lipids extracted from WWW and WWW‐NVs.

FAME	WWW (n = 5) (%relative quantification ± SD)	WWW‐NVs (n = 5) (%relative quantification ± SD)
14:0	7.16 ± 1.11	2.85 ± 1.48**
16:0	41.5 ± 0.72	35.68 ± 1.69**
16:1 9t	0.05 ± 0.02	0.08 ± 0.02*
16:1 6c (+7c)	0.19 ± 0.08	0.26 ± 0.06
16:1 9c	1.55 ± 0.04	1.24 ± 0.15*
17:0	0.66 ± 0.05	0.71 ± 0.13
18:0	12.49 ± 0.26	15.68 ± 1.16***
18:1 9t	0.54 ± 0.04	0.64 ± 0.08
18:1 11t	0.72 ± 0.04	0.62 ± 0.16
18:1 8c	0.97 ±0.50	0.95 ± 0.24
18:1 9c	26.88 ± 0.86	28.07 ± 3.21
18:1 11c	0.96 ± 0.12	1.51 ± 0.22*
18:2 5c8c	0.39 ± 0.02	0.32 ± 0.08*
18:2 9t12t	0.08 ± 0.02	0.20 ± 0.09*
18:2 9t12c	0.09 ± 0.03	0.21 ± 0.04*
18:2 9c12t	0.23 ± 0.03	0.13 ± 0.01**
18:2 9c12c	3.12 ± 0.07	6.01 ± 0.97**
18:3 omega6	0.19 ± 0.07	0.33 ± 0.12**
18:3 omega3	0.47 ± 0.08	0.62 ± 0.10*
MIXCLA	0.65 ± 0.07	0.97 ± 0.33*
20:0	0.24 ± 0.10	0.47 ± 0.23*
20:1	0.16 ± 0.05	0.22 ± 0.03*
20:2 omega6	0.19 ± 0.06	0
DGLA	0.19 ± 0.05	0.49 ± 0.07
ARA	0.26 ± 0.07	0.58 ± 0.12**
EPA	0	0.19 ± 0.05**
DPA	0	0.32 ± 0.12*
SFA	62.09 ± 1.04	55.45 ± 2.12**
MUFA	30.67 ± 1.01	32.82 ± 2.03
TOT *trans*	1.73 ± 0.14	1.90 ± 0.29
PUFA	4.83 ± 0.29	8.85 ± 1.06*
omega‐6	3.96 ± 0.23	7.40 ± 1.06*
omega‐3	0.47 ± 0.08	1.13 ± 0.27
SFA/MUFA	2.02 ± 0.1	1.69 ± 0.16

*Note*: Values were determined in µg/mL based on GC peak areas identified and calibrated with standard references (corresponding to >99% of the total chromatographic peaks). The results, expressed as percentages relative to the total amount of all identified peaks (relative quantification % ± standard deviation, SD), were obtained from the analyses of five samples. The table also shows the distribution of fatty acids grouped according to their unsaturation level, as well as the content of n‐3 and n‐6 PUFA and the trans geometrical isomers of unsaturated fatty acids. Statistical analysis was carried out comparing WWW versus WWW‐NVs using student's *t*‐test (*p ≤ 0.05, **p ≤ 0.001, ***p ≤ 0.0001, WWW vs. WWW‐NVs).

Abbreviations: ARA, arachidonic acid; DGLA, dihomo‐gamma‐linolenic acid; DPA, docosapentaenoic acid; EPA, eicosapentaenoic acid; MIXCLA, mix conjugated linoleic acid; MUFA, monounsaturated fatty acids; PUFA, polyunsaturated fatty acids; SFA, saturated fatty acids.

These findings suggest that vesicles isolated from dairy by‐products, as well as those derived from milk, may contain PUFAs, which are known precursors of bioactive lipid mediator. In this context, it is noteworthy that oxylipins, oxygenated lipid mediators derived from PUFAs and known for their anti‐inflammatory properties, have been previously identified in human milk–derived vesicles (Albiach‐Delgado et al. 2024). For this reason, the lipid component of NV membrane, and, in particular, their PUFA, may contribute not only to vesicle membrane structure but also represent a potential source of bioactive lipid species involved in intercellular communication (Sagini et al. 2018).

Finally, a qualitative analysis of the polar metabolite content of WWW‐NVs was performed. This analysis revealed that among the 47 annotated metabolites the most represented were carbohydrates, followed by amino acids, tricarboxylic acids, and their derivatives. As expected, lactose was the most represented carbohydrate, followed by other disaccharides and monosaccharides (Table S1).

### 3.3 Biological Effects Exerted by WWW‐NVs on THP‐1 Cells

**FIGURE 3:**
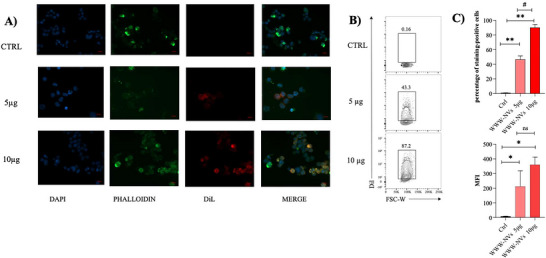
Internalization of WWW‐NVs by THP‐1 cells. (A) THP‐1 cells were exposed to DiL‐labeled NVs (5 and 10 µg proteins); after 2 h, the cells were fixed, actin filaments were stained with FITC‐labeled phalloidin, and nuclei were counterstained with DAPI. Magnification 40×. (B) Representative flow cytometry histograms showing the percentage of DiL‐labeled NV‐positive THP‐1 cells. (C) NV uptake expressed as the percentage of DiL‐positive cells and mean fluorescence intensity (MFI). The data are presented as mean ± SE of two independent experiments (*p < 0.05 vs. untreated cells; **p < 0.01 vs. untreated cells; #*p* < 0.05, WWW‐NVs 10 µg‐treated cells vs. WWW‐NVs 5 µg‐treated cells).

The experiments reported in this section aimed to evaluate the biological effects of WWW‐NVs on the human THP‐1 monocytic cell line, a well‐established in vitro model widely used to study the anti‐inflammatory and toxic properties of natural compounds (Zhao et al. 2019). To the best of our knowledge, the in vitro effects of vesicles derived from dairy by‐products have not yet been evaluated in human monocytic cell lines. The morphological and biochemical similarities between WWW‐NVs and milk‐derived vesicles suggest that WWW‐NVs might exert beneficial effects on mammalian cells, consistent with previous findings on milk‐derived vesicles (Salehi et al. 2024). The concentrations used in the experiments (2.5–10 µg of protein, corresponding to approximately 6 × 10^9^–1.5 × 10^1^ ^1^ particles, as estimated from NTA data) reported in this section were chosen based on previous studies reporting biological effects of milk‐derived EVs on THP‐1 cells (Izumi et al. 2015; Vahkal et al. 2025). First, we evaluated whether WWW‐NVs were efficiently internalized by THP‐1 cells. The uptake of WWW‐NVs by THP‐1 monocytes was assessed by evaluating the intracellular localization of DiL‐labeled WWW‐NVs using fluorescence microscopy and flow cytometry. Fluorescence microscopy images showed that WWW‐NVs were efficiently internalized by THP‐1 cells (Figure 3A). This result was confirmed by flow cytometry analysis, which demonstrated that WWW‐NVs were taken up by THP‐1 monocytes with approximately 40% and 80% efficiency at 5 and 10 µg proteins, respectively (Figure 3B). Histogram plots of the positive cell percentage and the mean fluorescence intensity suggest that a significantly higher number of THP‐1 monocytes took up WWW‐NVs, in a concentration‐dependent manner. In contrast, the number of vesicles taken up by each cell remains similar regardless of the WWW‐NVs concentration (Figure 3C).

**FIGURE 4:**
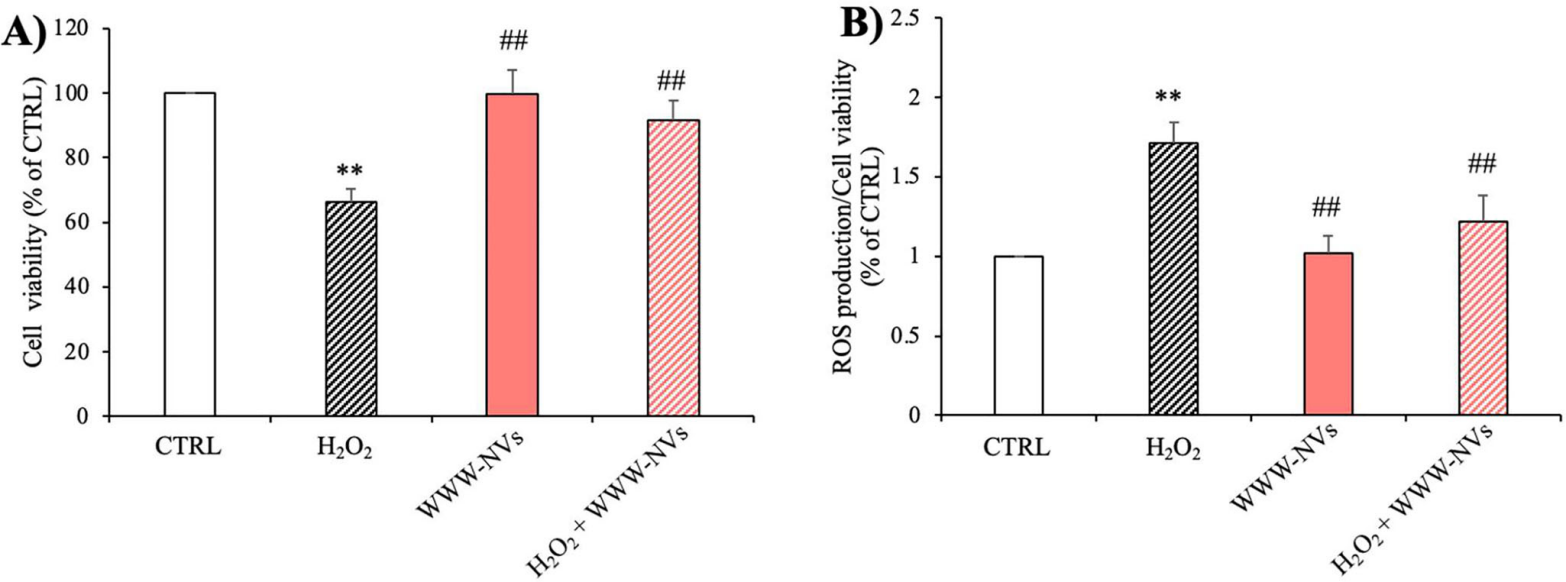
WWW‐NVs reduced H_2_O_2_‐induced cytotoxicity and ROS production. THP‐1 cells were co‐incubated for 24 h with H_2_O_2_ (250 µM) and WWW‐NVs (5 µg proteins). (A) Cell viability assessed by MTT assay. The percentage of live cells was calculated as the ratio between the absorbance of treated samples and controls (CTRL). (B) Intracellular ROS evaluated by DCFH‐DA assay. Fluorescence intensity was normalized to the values obtained from the MTT assay conducted in parallel; the results are presented as a percentage ratio compared to the CTRL. The data are reported as the mean ± SE of three independent experiments performed in triplicate (**p < 0.01 H_2_O_2_ cells vs. CTRL cells; ##*p* < 0.01 WWW‐NVs‐treated cells vs. H_2_O_2_‐treated cells; ##*p* < 0.01 WWW‐NVs+ H_2_O_2_‐treated cells vs. H_2_O_2_‐treated cells).

The cytoprotective and antioxidant properties of WWW‐NVs were evaluated in H_2_O_2_‐treated cells. THP‐1 cells were co‐incubated with WWW‐NVs (5 µg of proteins) and H_2_O_2_ (250 µM). The concentration of H_2_O_2_ used in these experiments was chosen based on preliminary experiments assessing the cytotoxicity of different H_2_O_2_ concentrations (data not shown). As reported in Figure 4, H_2_O_2_ enhanced significantly the intracellular ROS levels and reduced cell viability. Interestingly, the co‐incubation with WWW‐NVs significantly counteracted the cytotoxic effect (Figure 4A) and the intracellular ROS increase (Figure 4B) induced by H_2_O_2_. These data highlighted that WWW‐NVs protected THP‐1 cells from the cytotoxicity and increase of ROS levels induced by H_2_O_2_. This protective effect is consistent with observations reported for NVs derived from bovine and other species' milk against oxidative stress induced by different stimuli in intestinal epithelial cells (Wang et al. 2021; Gao et al. 2024) and in an animal model (Ibrahim et al. 2019).

**FIGURE 5:**
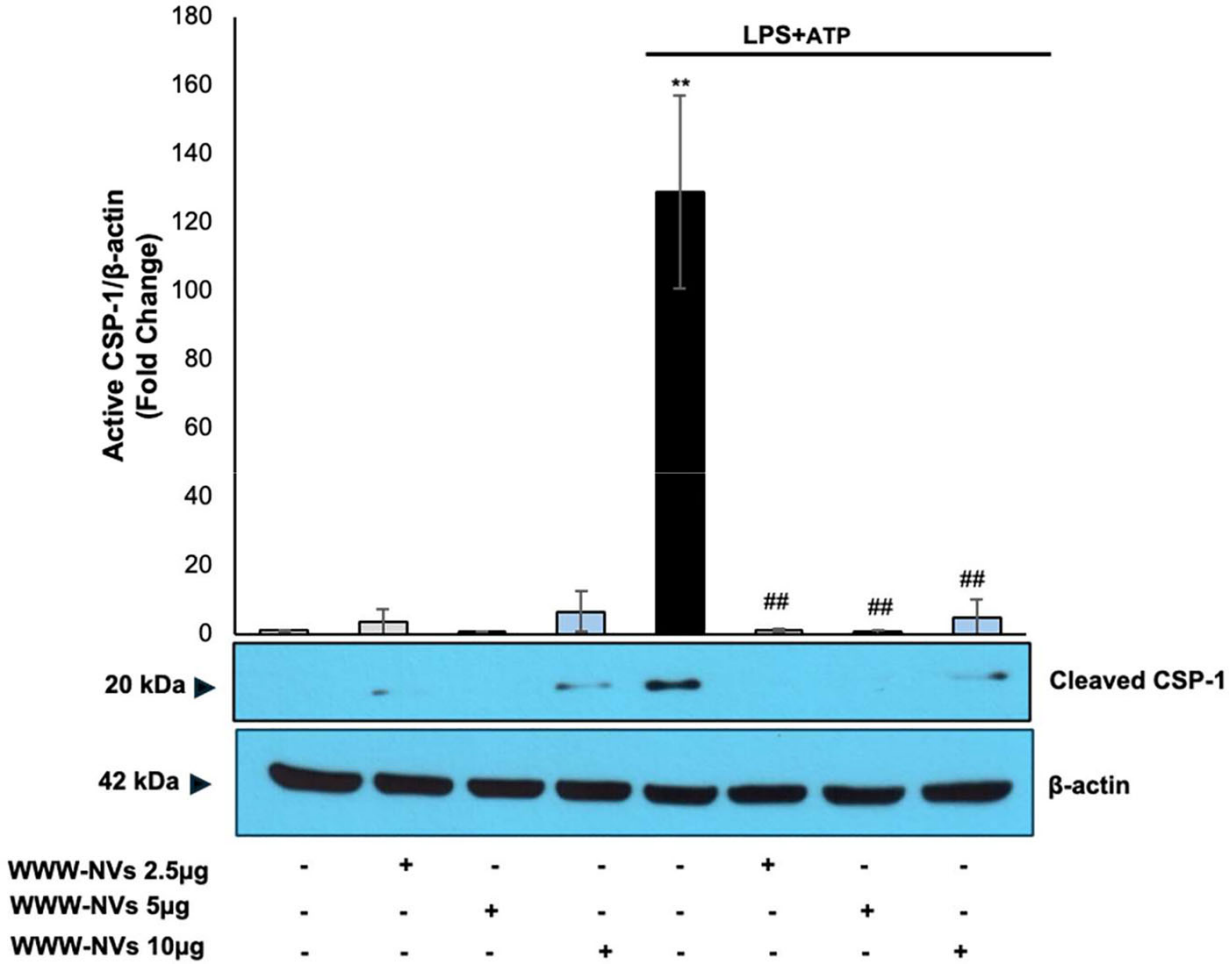
WWW‐NVs abrogate inflammasome activation in THP‐1 cells. THP‐1 cells were pre‐treated with different amounts of WWW‐NVs (2.5, 5, and 10 µg of proteins) for 1 h and subsequently primed with 10 µg/mL LPS for 20 min and then activated with 5 mM ATP for 40 min (LPS + ATP). Cell lysates were immunoblotted for caspase‐1. β‐actin was used as a loading control. Representative western blot images are shown. Histograms represent densitometric quantification and indicate the mean ± SE of at least three independent experiments (**p < 0.01 LPS‐treated cells vs. untreated cells; ##*p* < 0.01 WWW‐NVs‐treated cells vs. LPS+ATP treated cells).

The anti‐inflammatory properties of WWW‐NVs have been assessed by evaluating their effects on NLRP3‐inflammasome platform activation in human monocytic THP‐1 cells, a well‐established cell model used to study inflammasome activation induced by different stimuli (Mezzasoma et al. 2021; Mezzasoma et al. 2023; Campden et al. 2022). THP‐1 cells were cultured for 1 h with WWW‐NVs (2.5–10 µg proteins) before treatment with LPS+ATP. As shown in Figure 5, LPS+ATP treatment activated inflammasome, as indicated by the significantly elevated expression of the mature fragment p20 of the effector enzyme caspase‐1. The coincubation with WWW‐NVs was able to abrogate the LPS+ATP‐induced caspase‐1 activation (Figure 5), thus indicating an intrinsic anti‐inflammatory potential for WWW‐NVs. These findings are consistent with the immune‐modulatory and anti‐inflammatory properties attributed to milk‐derived EVs. In agreement with our findings, recent studies demonstrated that bovine milk EVs are incorporated in THP‐1 cells (Izumi et al. 2015) and that raw human and bovine milk EVs decreased the secretion of proinflammatory cytokines from LPS‐activated RAW264.7 cells (Ascanius et al. 2021).

Altogether, the results reported in this section indicate that WWW‐NVs possess intrinsic bioactivities, as they were efficiently internalized by THP‐1 cells and were able to induce cytoprotectective and anti‐inflammatory responses. In addition, our findings confirm that vesicles isolated from dairy by‐products may exert biological effects comparable to those of milk‐derived EVs, which are valued as drug delivery tools for their stability, ability to cross biological barriers and to carry bioactive molecules to target cells with low immunotoxicity (Ngu et al. 2023). In this study, the stability of WWW‐NVs under simulated gastrointestinal conditions has not been assessed. Previous studies on milk‐derived vesicles reported their stability in the digestive tract (Marsh et al. 2021), and we hypothesized that similar properties may be expected for NVs isolated from dairy by‐products, as it has been previously reported that cheesemaking whey EVs loaded with miRNAs and orally administered could cross the intestinal barrier and distribute to peripheral tissues (Del Saz‐Lara et al. 2025).

## 4 Conclusions

The results reported in this study demonstrated that NVs isolated from WWW, a byproduct generated during the industrial production of Grana Padano, share morphological and biochemical features previously reported to be associated with vesicles isolated from milk. WWW is an abundant byproduct that is collected and managed during cheese production processes. This byproduct is particularly attractive as a source of vesicles due to its routine production in large amount and low casein content (Del Saz‐Lara et al. 2025; Rocha‐Mendoza et al. 2021).

The isolation method used in this study is widely employed for the isolation of milk EVs (Caira et al. 2025; Özdemir 2020; Ross et al. 2021). We choose to use the NVs obtained with this method without additional purification steps (i.e., gradient centrifugation or size exclusion chromatography), as we detected the presence of vesicular protein markers associated with the NVs, that, along with a high PL/GL ratio, suggested an enrichment of the vesicular component in our NV preparations. However, in the absence of additional orthogonal purification steps, the potential co‐isolation of non‐vesicular components cannot be fully excluded. In addition, as also highlighted by Del Saz‐Lara et al. (2025), validating isolation methods (i.e., TFF and PEG precipitation) in addition to ultracentrifugation is required to address critical scalability challenges for pharmaceutical manufacturing.

Notably, we also demonstrated that WWW‐NVs were internalized and exerted biological effects (i.e., cytoprotective, antioxidant, and anti‐inflammatory) on THP‐1 monocyte, thus suggesting that these vesicles possess intrinsic bioactivities. Previous studies have demonstrated the feasibility of isolating NVs from dairy by‐products, specifically from acid whey derived from cottage cheese processing (Sukreet et al. 2021), and more recently EVs were isolated from whey fractions obtained after casein removal and subsequent milk acidification (Del Saz‐Lara et al. 2025). In this study, NVs were directly isolated from a dairy byproduct massively generated by industrial processing. To the best of our knowledge, this study also provides the first characterization of the lipid composition of NVs isolated from a dairy product, demonstrating that lipid composition of WWW‐NVs closely resembled that reported in literature for milk‐derived EVs. However, future studies will be necessary to directly compare the lipid composition of WWW‐NVs with that of NVs isolated from raw milk obtained from the same batch, to precisely define the impact of dairy processing on vesicle lipid profiles. In addition, the evaluation of the effects of WWW‐NVs on THP‐1 cells provides new insights into the functional properties of vesicles derived from dairy by‐products. As THP‐1 are a tumor‐derived cell line, future investigations are necessary to evaluate the biological effects exerted by WWW‐NVs on non‐tumoral cells, including intestinal epithelial cell models.

In conclusion, future studies are also needed to assess key parameters, including stability, targeting specificity, and immunotoxicity, which are crucial for the development of WWW‐NVs as nutraceutical tool and/or delivery vehicles for biomolecules and drugs. NV stability should be assessed by evaluating membrane integrity under conditions that mimic the gastrointestinal environment. Biochemical changes, with particular focus on lipid profiles, should also be analyzed in NVs incubated under these conditions.

As previously reported for milk‐derived EVs, cellular uptake and biological activity should be also evaluated in co‐culture systems, such as Caco‐2/HT29‐MTX or Caco‐2/THP‐1, which are vitro models of the intestinal microenvironment and intestinal inflammation (Roerig et al. 2021; Mecocci et al. 2022). Finally, future studies should define the optimal formulation strategies able to preserve the structural integrity and bioactivity of WWW‐NVs during long‐term storage at room temperature, taking into account the approaches currently used for milk‐derived EVs (Dogan et al. 2025). Altogether, these investigations are required to define the parameters required for the effective and safe use of WWW‐NVs in nutraceutical and delivery applications.

The valorization of WWW as a source of NVs represents a circular economy approach, enabling the recovery of high‐value bioactive components from a non‐food‐competing resource that is otherwise underutilized. Nevertheless, further investigations are required to optimize yields and evaluate process steps at an industrial scale, as well as to ensure the production of NVs that retain biophysical properties and bioactivity.

## Author Contributions

**Raffaella Latella**: investigation, formal analysis, writing – review and editing. **Anna Sansone**: investigation, writing – review and editing. **Carla Emiliani**: conceptualization, resources. **Carla Ferreri**: methodology, writing – review and editing. **Letizia Mezzasoma**: methodology, writing – review and editing. **Rita Romani**: methodology, writing – review and editing. **Marco Gargaro**: methodology, writing – review and editing. **Roberto Maria Pellegrino**: methodology, software. **Husam BR Alabed**: methodology, software. **Eleonora Calzoni**: methodology, writing – review and editing. **Stefano Giovagnoli**: methodology, writing – review and editing. **Luana Lugini**: methodology, writing – review and editing. **Cristina Federici**: methodology, writing – review and editing. **Federica Fratini**: methodology, writing – review and editing. **Lorena Urbanelli**: methodology, validation, visualization, writing – review and editing. **Sandra Buratta**: conceptualization, supervision, writing – review and editing, writing – original draft.

## Conflicts of Interest

The authors declare no conflicts of interest.

## Supporting information




**Supplementary Material**: jfds71243‐sup‐0001‐FigureS1.docx

**Supplementary Material**: jfds71243‐sup‐0002‐FigureS2.docx

**Supplementary Material**: jfds71243‐sup‐0003‐TableS1.docx
