# Supplementary material for: Characterization and Bioactivity of Nanovesicles Recovered From Industrial Cheesemaking Whey Wastewater
Source: J Food Sci. 2026 Jun 23;91(6):e71243. doi: 10.1111/1750-3841.71243 (PMC13288309; doi:10.1111/1750-3841.71243)
Supplement: Supplementary file 1 — Supplementary Material: jfds71243‐sup‐0001‐FigureS1.docx [file JFDS-91-0-s002.docx]

**
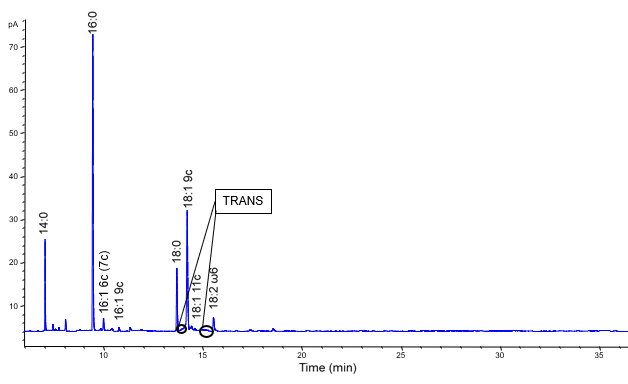
**

**A)**

**
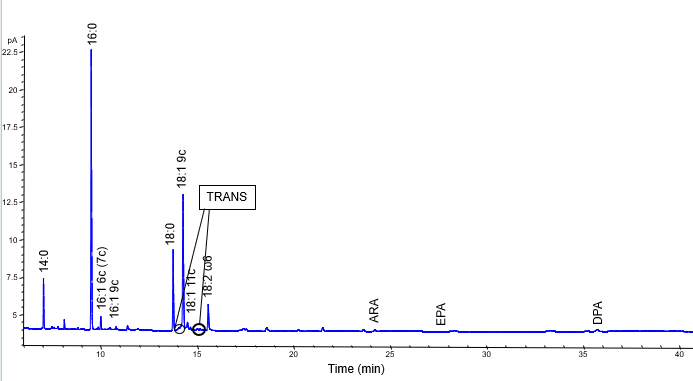
**

**B)**

**Figure S1: Representative GC-chromatogram of fatty acid methyl esters (FAME) obtained from WWW (A) and WWW-NVs (B).**
