# Supplementary material for: Characterization and Bioactivity of Nanovesicles Recovered From Industrial Cheesemaking Whey Wastewater
Source: J Food Sci. 2026 Jun 23;91(6):e71243. doi: 10.1111/1750-3841.71243 (PMC13288309; doi:10.1111/1750-3841.71243)
Supplement: Supplementary file 2 — Supplementary Material: jfds71243‐sup‐0002‐FigureS2.docx [file JFDS-91-0-s001.docx]

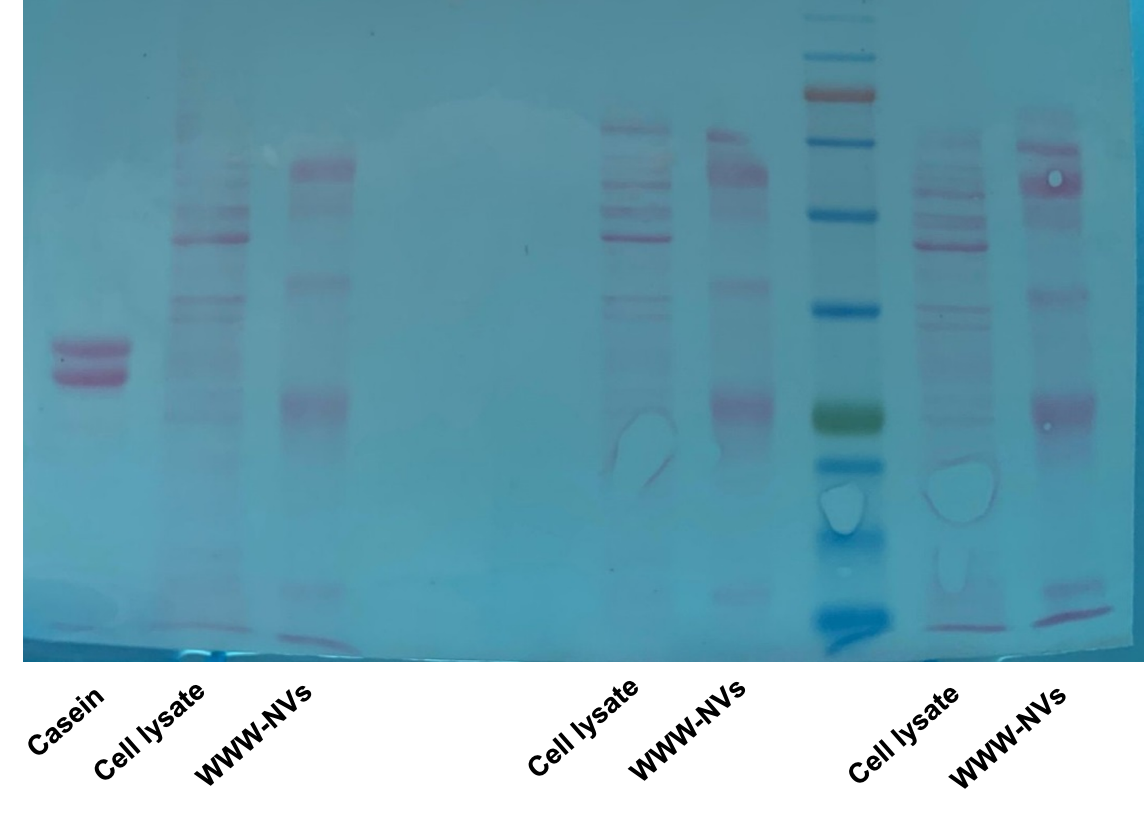


**Figure S2. PVDF membrane stained with Ponceau Red.** Casein (10 µg), BEAS-2B cell lysate (30 µg) and WWW-NVs (30 µg) were separated onto 10 % SDS-PAGE and transferred on PVDF membrane.
