# Supplementary material for: Characterization and Bioactivity of Nanovesicles Recovered From Industrial Cheesemaking Whey Wastewater
Source: J Food Sci. 2026 Jun 23;91(6):e71243. doi: 10.1111/1750-3841.71243 (PMC13288309; doi:10.1111/1750-3841.71243)
Supplement: Supplementary file 3 — Supplementary Material: jfds71243‐sup‐0003‐TableS1.docx [file JFDS-91-0-s003.docx]

**Supporting Information**

**Table S1:** **Polar metabolites ordered by area annotated in WWW-NVs.**

| **WWW-NVs** | | |
| --- | --- | --- |
|  | **Name** | **Peak Area**  (Media; n=4) |
| 1  2  3  4  5  6  7  8  9  10  11  12  13  14  15  16  17  18  19  20  21  22  23  24  25  26  27  28  29  30  31  32  33  34  35  36  37  38  39  40  41  42  43  44  45  46  47 | Lactose  4-O-.beta.-Galactopyranosyl-D-mannopyranose  D-Fructose  6.alpha.-Mannobiose  Erucamide  3.alpha.-Mannobiose  1-Stearoyl-2-linoleoyl-sn-glycero-3-phospho-L-serine  .alpha.,.beta.-Trehalose  1-Palmitoyl-3-oleoyl-sn-glycero-2-phosphoethanolamine  Citric acid  1-Stearoyl-2-linoleoyl-sn-glycero-3-phosphoethanolamine  2-Oleoyl-1-stearoyl-sn-glycero-3-phosphoserine  N-Acetyl-D-galactosamine-6-phosphate  trans-Aconitic acid  Melibiose  Populoside  Betaine  1-Palmitoyl-2-linoleoyl-sn-glycero-3-phosphocholine  2,3-Dihydroxypropyl dihydrogen phosphate  2-Linoleoyl-1-palmitoyl-sn-glycero-3-phosphoethanolamine  1-Palmitoyl-2-myristoyl-sn-glycero-3-phosphocholine  1-Stearoyl-2-myristoyl-sn-glycero-3-phosphocholine  .alpha.-D-Mannose 1-phosphate  1-Oleoyl-2-myristoyl-sn-glycero-3-phosphocholine  5.alpha.-Cholest-7-en-3.beta.-ol  L-Leucine  1-Oleoyl-2-palmitoyl-sn-glycero-3-phosphocholine  2-Oleoyl-1-palmitoyl-sn-glycero-3-phosphoserine  Pro-Gln  3.alpha.,6.alpha.-Mannotriose  Palmitoyl sphingomyelin  6'-Sialyllactose  D-2-Phosphoglyceric acid  1,2-Dipentadecanoyl-sn-glycero-3-phosphocholine  Cholesterol 3-sulfate  L-Carnitine  1-Oleoyl-sn-glycero-3-phosphoethanolamine  2-Oleoyl-1-palmitoyl-sn-glycero-3-phosphocholine  1,2-Ditetradecanoyl-sn-glycero-3-phosphocholine  Creatinine  Maltose  1,2-Dioleoyl-sn-glycero-3-phosphoethanolamine  Glycerophosphocholine  Acetyl-L-carnitine  DL-Ornithine  N-Lauroyl-L-arginine  Uric acid | 1.22x10^8^  1.92 x10^7^  6.06 x10^6^  5.07 x10^6^  3.58 x10^6^  3.22 x10^6^  2.46 x10^6^  2.23 x10^6^  2.15 x10^6^  2.11 x10^6^  2.07 x10^6^  2.04 x10^6^  1.83 x10^6^  1.76 x10^6^  1.53 x10^6^  1.22 x10^6^  1.20 x10^6^  1.12 x10^6^  1.09 x10^6^  1.07 x10^6^  1.02 x10^6^  8.51 x10^5^  6.29 x10^5^  6.21 x10^5^  6.18 x10^5^  5.73 x10^5^  5.37 x10^5^  5.21 x10^5^  3.73 x10^5^  3.55 x10^5^  3.25 x10^5^  2.75 x10^5^  2.63 x10^5^  2.61 x10^5^  2.47 x10^5^  2.31 x10^5^  1.99 x10^5^  1.87 x10^5^  1.60 x10^5^  1.49 x10^5^  1.43 x10^5^  1.28 x10^5^  1.10 x10^5^  1.07 x10^5^  3.95 x10^4^  3.60 x10^4^  3.42 x10^4^ |
